# SARCOPENIA AND MOBILITY DYSFUNCTION AS INDICATORS OF INCREASED ADRD RISK IN ASYMPTOMATIC DISEASE

**DOI:** 10.1093/geroni/igad104.1273

**Published:** 2023-12-21

**Authors:** Magdalena Tolea, Lilah Besser, James Galvin

**Affiliations:** University of Miami Miller School of Medicine, Miami, Florida, United States; University of Miami, Miami, Florida, United States; University of Miami, Miami, Florida, United States

## Abstract

Sarcopenia, the age-related loss of muscle mass and function, affects up to 30% of older adults. In addition to being an established component of frailty and predictor of mobility dysfunction, sarcopenia is associated with cognitive decline and increased risk of cognitive impairment, with ~70% individuals with late-stage dementia having sarcopenia. In this study, we investigated whether sarcopenia differentiates high- vs low- ADRD risk among 141 community-dwelling, older adults who were healthy controls (HC) or had mild cognitive impairment (MCI) (65% female; mean Age=70±11yr; mean Educ=16±3yr). Using the Brain Health Platform (BHP) developed at the Comprehensive Center for Brain Health, we categorized participants based on measures of resilience, vulnerability, cognitive performance as: Low-risk HC (36.9%); High-risk HC (12.8%), and MCI (50.4%). Amyloid positivity increased across risk groups (20% in Low-risk HC; 33.3% in High-risk HC; 39.1% in MCI). Prevalence of sarcopenia was 26.7% and increased by ADRD risk (12.2% vs 18.8% vs 39.1%, p=0.004). The MCI group had lower flexibility compared to both the low-risk (p=0.024) and high-risk HC (p=0.039) groups. Timed Up and Go (TUG) score increased with risk group (p< 0.001MCI vs Low-risk HC; p=0.005MCI vs High-risk HC). Both mobility measures (i.e., flexibility, TUG) showed good discrimination for MCI vs Low-risk HC [AUC=0.77flexibility; AUC=0.78TUG] as well as for MCI vs High-risk HC [AUC=0.71flexibility; AUC=0.73TUG). Findings suggest that sarcopenia and other markers of mobility dysfunction have the potential to identify asymptomatic individuals at increased risk of ADRD, who could benefit from early intervention.

